# Supplementary material for: The unquantified mass loss of Northern Hemisphere marine-terminating glaciers from 2000–2020
Source: Nat Commun. 2022 Oct 11;13:5835. doi: 10.1038/s41467-022-33231-x (PMC9553960; doi:10.1038/s41467-022-33231-x)
Supplement: Supplementary file 1 — Supplementary Information [file 41467_2022_33231_MOESM1_ESM.docx]

**Supplementary Materials**

**Supplemental Methods**

***Systematic errors: debiasing***

We compared two independent measurements or modelled values of ice velocity and ice thickness to infer biases and associated spatial correlations of the uncertainties. Biases are global systematic errors that do not depend on the spatial distance between observations. Such biases affect the variance and are therefore corrected prior to further spatial analysis. Errors that occur at the local or regional scale, including errors potentially introduced during debiasing, are reported as long-range correlation in our analysis. Our comparison of two independent data sources is a proxy for identifying correlated error to the unknown ground truth, which we expect to yield conservative uncertainties as it combines sources of errors from either two measurements, or that of a measurement and a model.

For velocity, we subtracted all measured ITS_LIVE velocities with measured MEaSUREs velocities acquired in the same year and at the same flux gate points. To mitigate the effect of data outliers on our analysis of variance, we excluded differences larger than five times the normalized median absolute deviation. We found no statistically significant bias between the datasets, and therefore assume that velocity estimates are unbiased at the global-scale.

For ice thickness, we subtracted all measurements from modelled values. Those sources of ice thickness were all adjusted for time differences to the year 2005 for the decade 2000-2010, or 2015 for 2010-2020, based on decadal elevation change rates extracted at the same locations. Measurements without a date were timestamped to 2000. We excluded differences between the measured and modelled values that were larger than five times the normalized median absolute deviation. We found a significant bias between measured and modelled ice thickness values of 134.5 m globally, due to overestimation by the model. This bias was nearly consistent between all RGI regions, ranging from 110 to 145 m (excluding Alaska, which contained fewer points of comparison). Modelled ice thickness provided a good explanatory variable for this bias, so it was used to correct the model estimates at each flux gate or terminus area using a linear relationship (Supplemental Fig. 6). After debiasing, the remaining difference between modelled and measured ice thickness was 1.7 m globally, and -35 to 45 m regionally.

**Random errors: spatial correlations**

We estimated spatial correlations by deriving empirical variograms. We used the semi-variance estimator by Matheron^70^ with random pairwise subsampling using at most 10,000 samples^69^. We validated the decorrelation of the variograms at long-ranges (>5000 km) by comparing their amplitude to the global variance of the differences, estimated by the square of the normalized median absolute deviation. We then standardized the variograms by long-range variances (>2000 km) to express them as a percentage of the global variance. For ice thickness, the global variance corresponded on average to a standard deviation of ± 65 m, after debiasing. For velocity, it corresponded on average to ± 20 m a^-1^. Given the variability of velocity and ice thickness uncertainties across glaciers and regions, our standardization assumes a consistency of the spatial correlations as a percentage of variance for glaciers and regions, supported by a good agreement of standardized spatial correlation subsampled independently between regions.

We modelled our empirical variograms of velocity and ice thickness by a sum of three spherical models optimized by least-squares:

$C(l)=\sum_{i=1}^{3} \gamma_{i}(l, r_{i}, s_{i})$(S1)

where l is the spatial lag (i.e. distance between observations) and $\gamma$ is the spherical variogram model:

$\gamma(l,r,s)=s\left( 1.5\frac{l}{r}-0.5\frac{l^{3}}{r} \right)$if $l<r$, otherwise $\gamma=s$,

with $r$ the range (i.e. correlation length) and $s$ the partial sill (i.e. correlated variance).

To estimate the spatial correlation in ice discharge at the flux gate (Equation 8), we combined the variograms of velocity and ice thickness uncertainties, as it was not possible to sample enough locations containing all four ITS_LIVE velocity, MEaSUREs velocity, measured ice thickness, and modelled ice thickness, to directly determine a variogram for ice discharge differences. Consequently, we approximated the ice discharge variogram for each glacier and region by an independent product of variograms:

$C_{\dot{D}_{gate}}(l)=\frac{\left[ C_{V}(l)\cdot\left( \underline{\sigma_{V}}\cdot\underline{H}\cdot\underline{d} \right)^{2}+C_{H}(p)\cdot\left( \underline{V}\cdot\underline{\sigma_{H}}\cdot\underline{d} \right)^{2} \right]}{\left( \underline{\sigma_{V}}\cdot\underline{H}\cdot\underline{d} \right)^{2}+\left( \underline{V}\cdot\underline{\sigma_{H}}\cdot\underline{d} \right)^{2}}$ (S2)

We note that, conservatively, we applied the spatial correlations based on the comparison to ice thickness model estimates at locations where measurements exist, while measurement errors should be less correlated.

**Supplemental figures, table, and dataset**

**
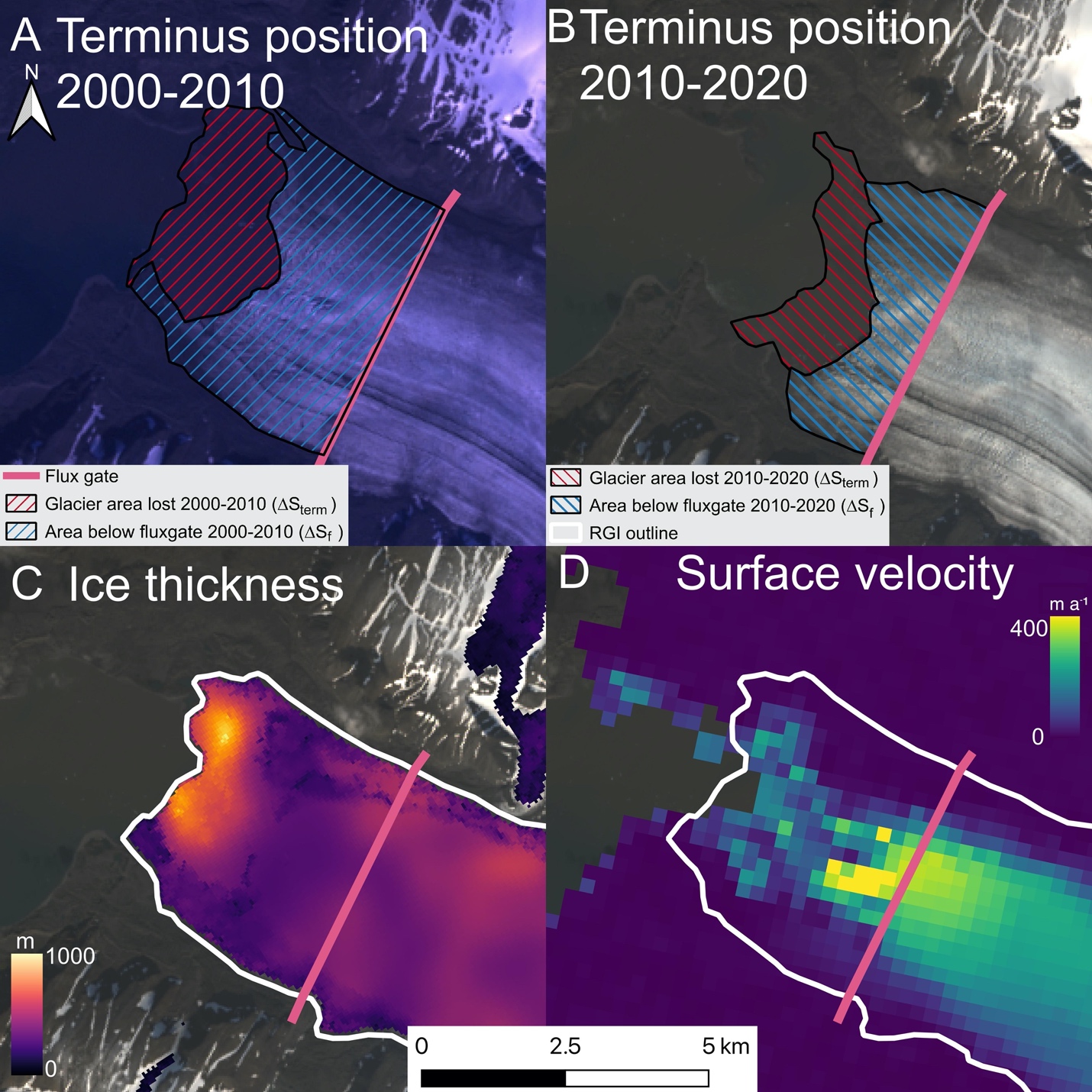
**

**Supplementary Figure 1**

Data sets used to compute frontal ablation exemplified for a glacier on Novaya Zemlya, Russia (75.2°N, 57.1°E). A) Glacier termini positions and glacier area lost by retreat or gained by advance (red hatching, ${\Delta S}_{term}$ in eq. 3) and area not involved in retreat/advance below the fluxgate (blue hatching, $S_{f}$ in eq. 2) for 2000-2010. B) Same as Fig S1A except for 2010-2020. C) Glacier thickness distribution derived from model data generated by Millan et al^53^. D) Surface velocity data for 2018 from ITS_LIVE^36^. Thickness ($H_{n}$) and velocity ($V_{n}$) data along the flux gate are used in eq. 2. Thickness data averaged over the most extended glacier terminus position during the considered period ($\overline{H}$) is used in eq. 3.

**
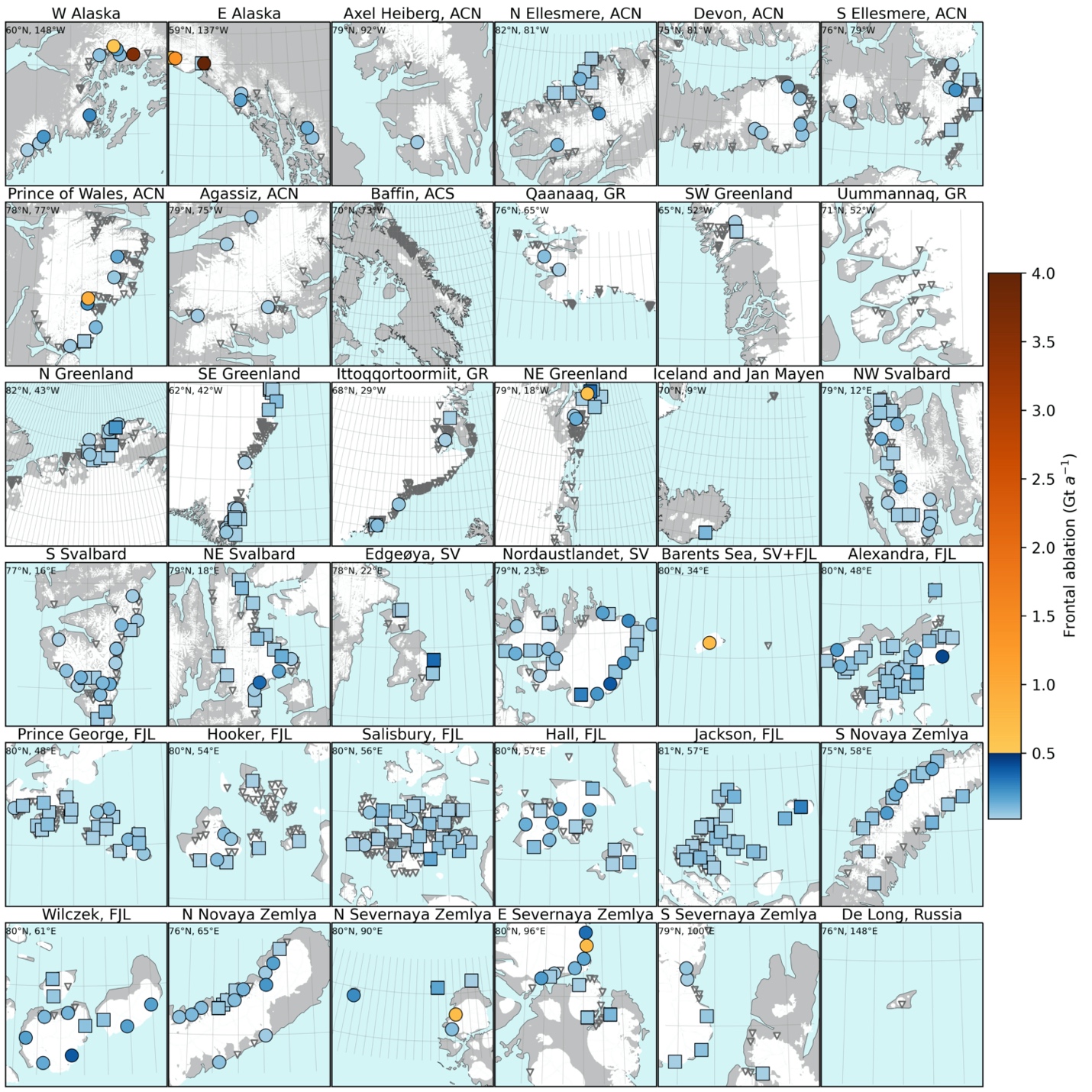
Supplementary Figure 2**

Frontal ablation by glacier for 2000-2010. Dark gray triangles indicate glaciers that have a frontal ablation less than 0.02 Gt a^-1^ (not shown in color bar) and squares indicate glaciers where the uncertainty is more than 50% of total frontal ablation. Glacierized area is marked in white. Latitude and longitude in top left of each panel indicate the center point of that panel and the grid is 1° by 1° for all panels, with 1 degree of latitude equal to 111 km. The only glacier off the color bar scale is Hubbard Glacier (4.05 Gt a^-1^; E Alaska). For uncertainties see Fig. S4. Abbreviations: ACN is Arctic Canada North, ACS is Arctic Canada South, GR is Greenland Periphery, SV is Svalbard, FJL is Franz Josef Land, NZ is Novaya Zemlya, SZ is Severnaya Zemlya; other abbreviations are cardinal directions.

**
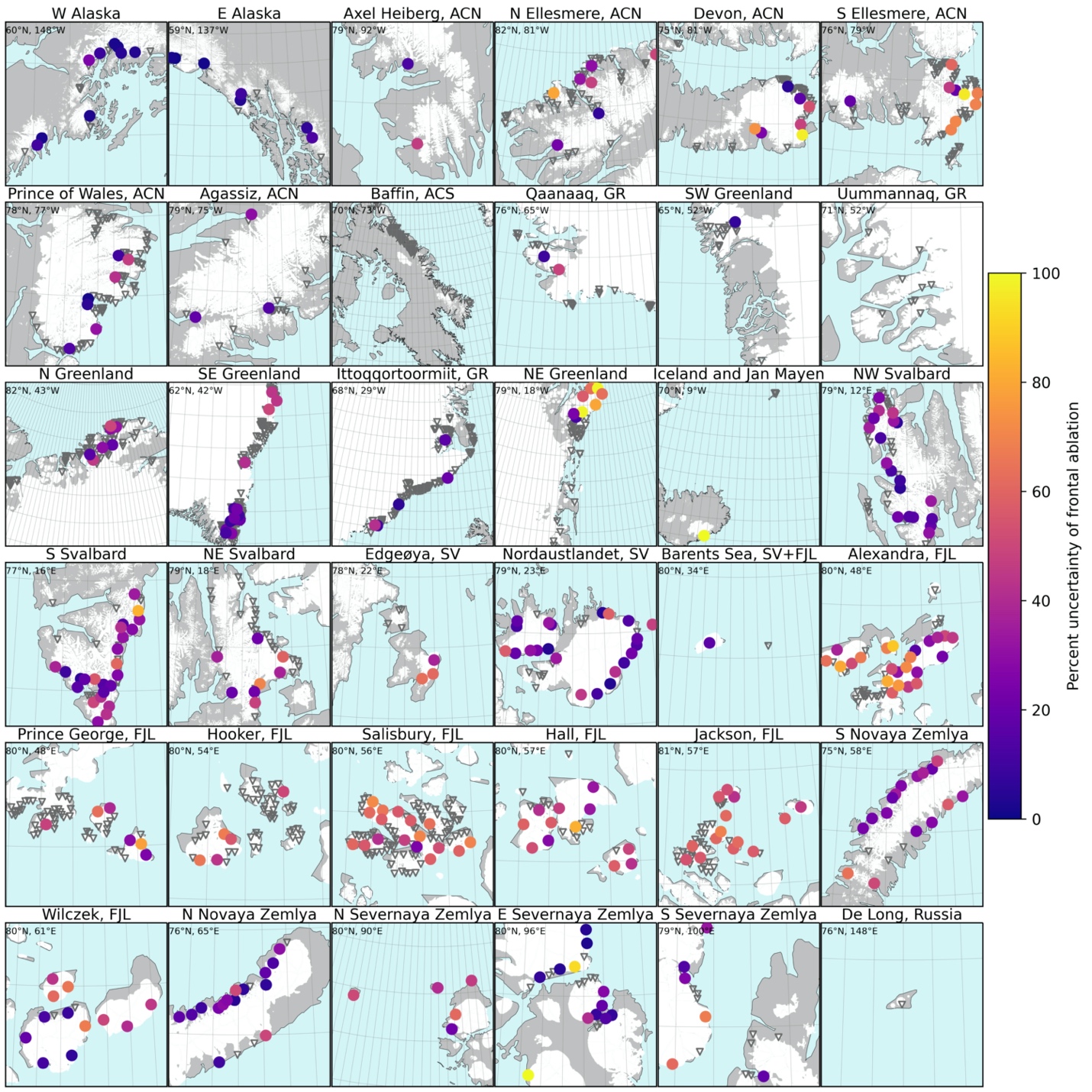
**

**Supplementary Figure 3**

Frontal ablation uncertainties by glacier for 2010-2020. Colored dots correspond to the uncertainty of the frontal ablation calculation from 0% (cool colors) to 100% (warm colors) of the total shown in Fig. 2. Grey triangles indicate glaciers that have a frontal ablation of less than 0.02 Gt a^-1^. Grid is 1° by 1° for all subplots and the coordinates in the top left of each subplot shows the center latitude and longitude of that subplot. Abbreviations: ACN is Arctic Canada North, ACS is Arctic Canada South, GR is Greenland Periphery, SV is Svalbard, FJL is Franz Josef Land, NZ is Novaya Zemlya, SZ is Severnaya Zemlya; other abbreviations are cardinal directions.

**
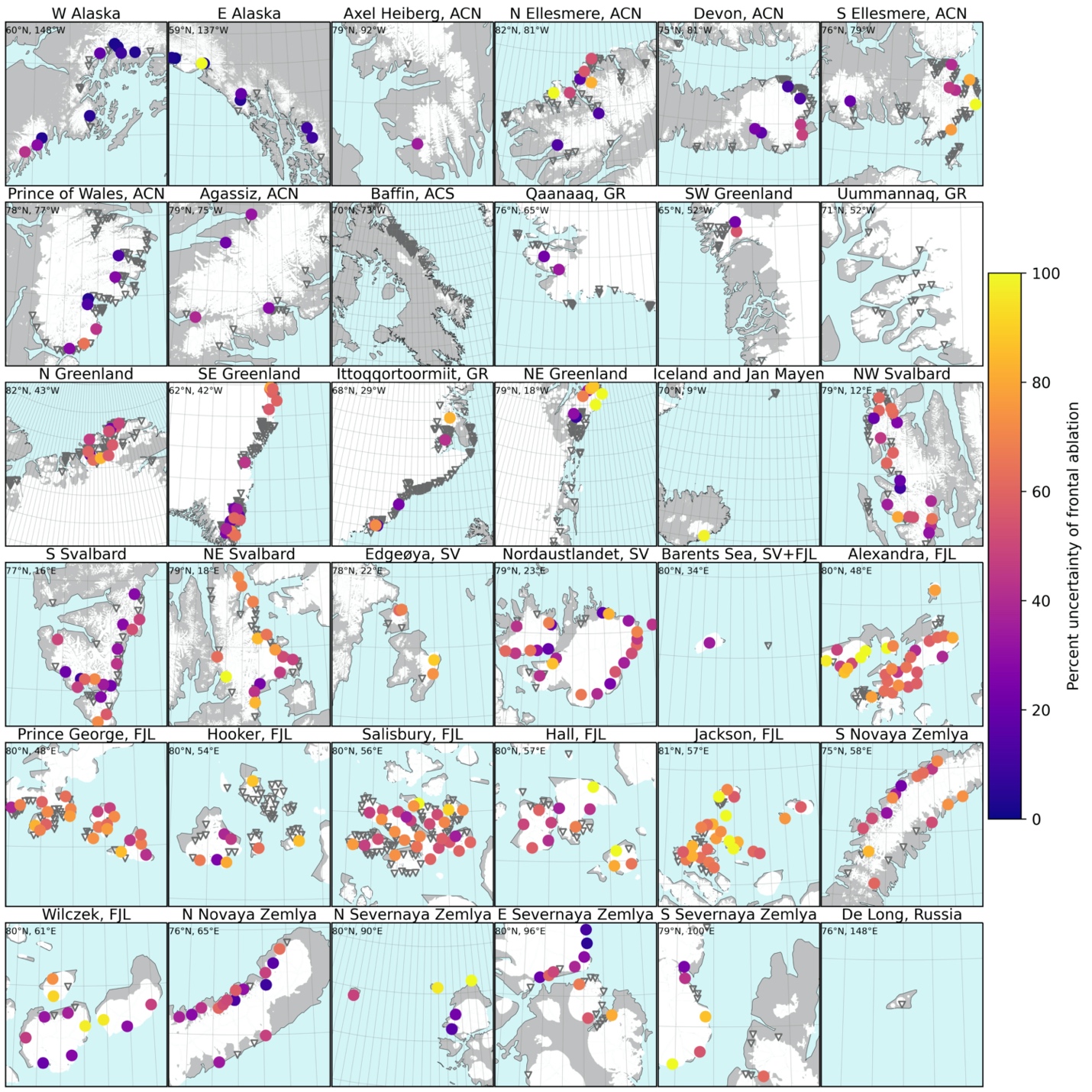
**

**Supplementary Figure 4**

Frontal ablation uncertainties by glacier for 2000-2010. Colored dots correspond to the uncertainty of the frontal ablation calculation from 0% (cool colors) to 100% (warm colors) of the total shown in Supplementary Fig. 2. Grey triangles indicate glaciers that have a frontal ablation of less than 0.02 Gt a^-1^. Grid is 1° by 1° for all subplots and the coordinates in the top left of each subplot shows the center latitude and longitude of that subplot. Abbreviations: ACN is Arctic Canada North, ACS is Arctic Canada South, GR is Greenland Periphery, SV is Svalbard, FJL is Franz Josef Land, NZ is Novaya Zemlya, SZ is Severnaya Zemlya; other abbreviations are cardinal directions.

**
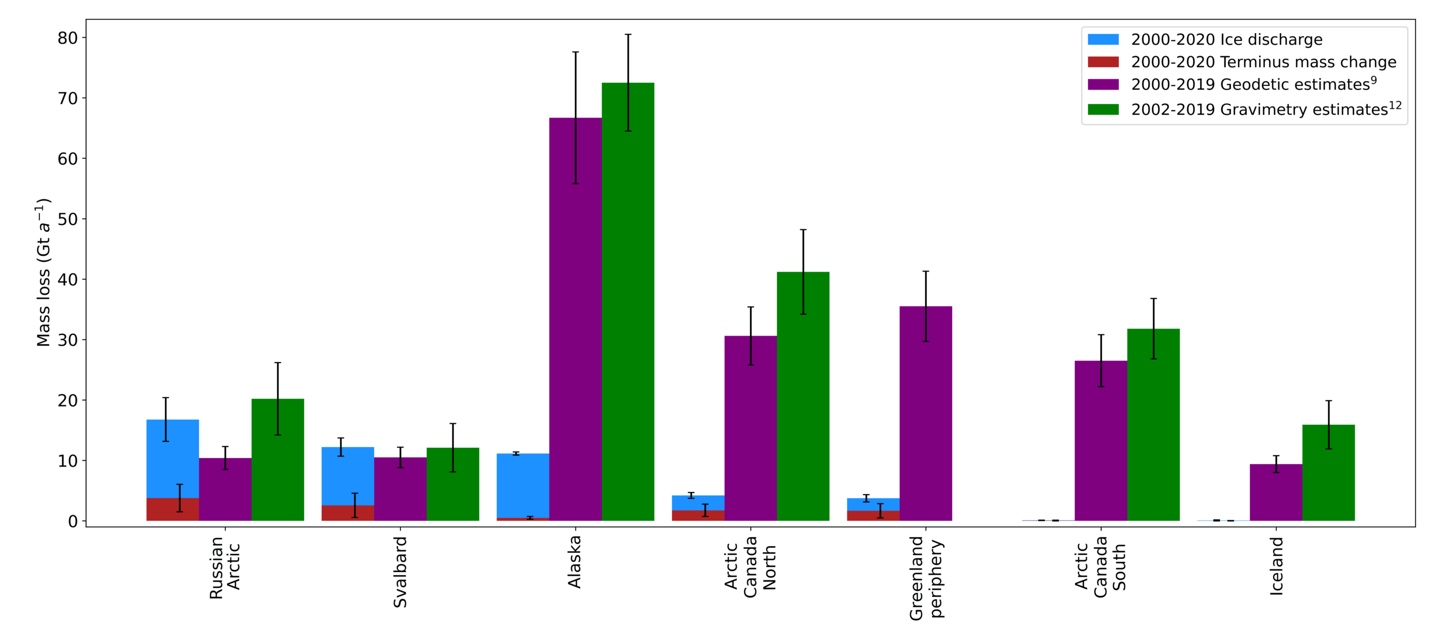
**

**Supplementary Figure 5**

Mass loss by region and method (frontal ablation, geodetic or gravimetric). Frontal ablation results from this study are shown in blue (ice discharge) and red (terminus mass change) as an annual mean over 2000-2020, along with regional mass loss estimates from geodetic methods^9^ and gravimetry^12^ (excluding Greenland Periphery).

**Supplementary Figure 6.**Debiasing of modelled ice thickness estimates. Bias between modelled and measured ice thicknesses after adjustment of surface elevations to 2005 (for 2000-2010 period) or 2015 (for 2010-2020 period) to account for geodetic mass balance. The bias is empirically estimated by binning the difference of measurement and model estimates within 20 intervals (every 40 m) of modelled ice thickness. The sample count of each bin represents the number of flux gate points where measured and modelled ice thickness were compared. The bias dependency to modelled ice thickness is constrained by a linear function optimized by least-squares. The bias is later removed by subtracting the value of this function at each location.

**Supplementary Figure 7.**

Spatial correlation of velocity estimates. Empirical and modelled variogram of velocity estimates, expressed as a percentage of the global velocity variance. The empirical variogram is estimated from the differences between ITS_LIVE and MEaSUREs velocities in Greenland compared at the same locations and for the same yearly periods. The sample count describes the number of pairwise flux gate comparisons used to estimate the variogram at each spatial lag (i.e. distance between observations). The modelled variogram is a sum of three spherical models optimized by least-squares (Equation S1). The first spherical model with range (correlation length) of 0.7 km has a partial sill (correlated variance) of 63% of the global velocity variance, the second model with range 18 km of 26% of the variance, and the third model with range 1,040 km of 11% of the variance. The spatial correlation of velocity is deduced by subtracting the modelled variogram to the global variance, and used to propagate uncertainties from pixel to larger scales in ice discharge estimates.

**Supplementary Figure 8.**

Spatial correlation of ice thickness estimates. Empirical and modelled variogram of ice thickness estimates, expressed as a percentage of the global ice thickness variance. The empirical variogram is estimated from the differences between measured and de-biased modelled ice thicknesses compared at the same locations, and adjusted to the same year with elevation changes. The sample count describes the number of pairwise flux gate comparisons used to estimate the variogram at each spatial lag (i.e. distance between observations). The modelled variogram is a sum of three spherical models optimized by least-squares (Equation S1). The first spherical model with range (correlation length) of 2 km has a partial sill (correlated variance) of 27% of the global ice thickness variance, the second model with range 47 km of 50% of the variance, and the third model with range 1,404 km of 23% of the variance. The spatial correlation of ice thickness is deduced by subtracting the modelled variogram to the global variance, and used to propagate uncertainties from pixel to larger scales in both ice discharge and terminus mass change estimates.

|  | **Frontal ablation 2000-2020** | **Climatic mass balance correction for discharge** | | **Area for climatic mass balance correction for discharge** | | **Climatic mass balance correction for terminus change** | | **Area for climatic mass balance correction for terminus change** | | **Marine-terminating glacier area** | **Total glacier area^17^** | **Submarine-frontal ablation 2000-2020** | **Geodetic mass loss^9^** | **Corrected, total Geodetic mass loss** |
| --- | --- | --- | --- | --- | --- | --- | --- | --- | --- | --- | --- | --- | --- | --- |
|  |  | **2000-2010** | **2010-2020** | **2000-2010** | **2010-2020** | **2000-2010** | **2010-2020** | **2000-2010** | **2010-2020** |  |  |  |  |  |
|  | **(Gt a^-1^)** | **(Gt a^-1^)** | **(Gt a^-1^)** | **(km^2^)** | **(km^2^)** | **(Gt a^-1^)** | **(Gt a^-1^)** | **(km^2^)** | **(km^2^)** | **(km^2^)** | **(km^2^)** | **(Gt a^-1^)** | **(Gt a^-1^)** | **(Gt a^-1^)** |
| **Alaska** | 11.14 ± 0.36 | -1.10 ± 0.22 | -0.94 ± 0.17 | 134.2 | 96.2 | -0.08 ± 0.05 | -0.18 ± 0.06 | 38.6 | 46.7 | 12000 | 87000 | 0.34 ± 0.18 | 66.7 ± 10.9 | 67.0 ± 10.9 |
| **Arctic Canada North** | 4.21 ± 1.15 | -2.00 ± 0.32 | -2.49 ± 0.39 | 1511.6 | 1514.3 | -0.20 ± 0.07 | -0.23 ± 0.07 | 312.1 | 263.1 | 48000 | 105000 | 1.22 ± 0.75 | 30.6 ± 4.8 | 31.8 ± 4.9 |
| **Arctic Canada South** | 0.09 ± 0.08 | -0.11 ± 0.02 | -0.09 ± 0.01 | 39.3 | 31.9 | -0.01 ± 0.01 | -0.01 ± 0.01 | 9.5 | 7.5 | 3100 | 41000 | 0.04 ± 0.04 | 26.5 ± 4.3 | 26.5 ± 4.3 |
| **Greenland Periphery** | 3.74 ± 1.33 | -1.66 ± 0.31 | -1.72 ± 0.32 | 1218.7 | 1099.4 | -0.26 ± 0.08 | -0.17 ± 0.07 | 387.5 | 189.9 | 29000 | 90000 | 1.17 ± 0.91 | 35.5 ±5.8 | 36.7 ± 5.9 |
| **Iceland** | 0.06 ± 0.06 | -0.21 ± 0.04 | -0.18 ± 0.04 | 27.1 | 23.7 | -0.01 ± 0.0 | -0.01 ± 0.0 | 2.3 | 3.4 | 1100 | 11000 | 0.01 ± 0.01 | 9.4 ± 1.4 | 9.4 ± 1.4 |
| **Svalbard and Jan Mayen** | 12.22 ± 2.57 | -1.90 ± 0.31 | -1.88 ± 0.32 | 1841.7 | 1689.9 | -0.15 ± 0.05 | -0.22 ± 0.08 | 355.6 | 519.9 | 23000 | 34000 | 1.80 ± 1.52 | 10.5 ± 1.7 | 12.3 ± 2.3 |
| **Russia: Franz Josef Land** | 8.95 ± 4.17 | -1.04 ± 0.2 | -1.31 ± 0.25 | 1789.8 | 2019.0 | -0.10 ± 0.04 | -0.13 ± 0.05 | 326.2 | 354.4 | 11000 | 52000* | 1.55 ± 1.32 | 10.4 ± 1.9* | 13.0 ± 2.9* |
| **Russia: Novaya Zemlya** | 3.41 ± 0.98 | -0.56 ± 0.08 | -0.47 ± 0.07 | 387.9 | 257.5 | -0.09 ± 0.02 | -0.13 ± 0.02 | 124.7 | 135.9 | 14000 |  | 0.58 ± 0.52 |  |  |
| **Russia: Severnaya Zemlya** | 4.42 ± 1.01 | -0.79 ± 0.16 | -0.89 ± 0.2 | 1207.4 | 1469.6 | -0.01 ± 0.01 | -0.05 ± 0.04 | 87.7 | 369.5 | 8000 |  | 0.51 ± 0.35 |  |  |
| **Total** | 48.23 ± 5.43 | -9.38 ± 0.64 | -9.97 ± 0.71 | 8157.7 | 8201.5 | -0.92 ± 0.14 | -1.13 ± 0.15 | 1644.3 | 1890.4 | 150000 | 420000 | 7.22 ± 2.80 | 189.6 ± 14.2 | 196.8 ± 14.5 |

**Table S1.**

Components of mass loss summarized by region. For details on individual glaciers see dataset S1.

*Indicates regional sum for Russia rather than subregions.

**Dataset S1.**

Raw data used to calculate the calving flux for every glacier. See table header and Materials and Methods section for more details.
