# Supplementary material for: The unquantified mass loss of Northern Hemisphere marine-terminating glaciers from 2000–2020
Source: Nat Commun. 2022 Oct 11;13:5835. doi: 10.1038/s41467-022-33231-x (PMC9553960; doi:10.1038/s41467-022-33231-x)
Supplement: Supplementary file 3 — Description of Additional Supplementary Files [file 41467_2022_33231_MOESM3_ESM.pdf]

Description of Additional Supplementary Files

File Name: Supplementary Data 1
Description: Raw data used to calculate the calving flux for every glacier. See table header and Materials and Methods section for more details.
